# Interactions among Redox Regulators and the CtrA Phosphorelay in *Dinoroseobacter shibae* and *Rhodobacter capsulatus*

**DOI:** 10.3390/microorganisms8040562

**Published:** 2020-04-14

**Authors:** Sonja Koppenhöfer, Andrew S. Lang

**Affiliations:** Department of Biology, Memorial University of Newfoundland, St John’s, NL A1B 3X9, Canada; skoppenhofer@mun.ca

**Keywords:** Alphaproteobacteria, Rhodobacteraceae, nitric oxide, quorum sensing, gene transfer agent, motility, Crp/Fnr, Dnr, RegA, ChpT

## Abstract

Bacteria employ regulatory networks to detect environmental signals and respond appropriately, often by adjusting gene expression. Some regulatory networks influence many genes, and many genes are affected by multiple regulatory networks. Here, we investigate the extent to which regulatory systems controlling aerobic–anaerobic energetics overlap with the CtrA phosphorelay, an important system that controls a variety of behavioral processes, in two metabolically versatile alphaproteobacteria, *Dinoroseobacter shibae* and *Rhodobacter capsulatus*. We analyzed ten available transcriptomic datasets from relevant regulator deletion strains and environmental changes. We found that in *D. shibae*, the CtrA phosphorelay represses three of the four aerobic–anaerobic Crp/Fnr superfamily regulator-encoding genes (*fnrL*, *dnrD*, and especially *dnrF*). At the same time, all four Crp/Fnr regulators repress all three phosphorelay genes. Loss of *dnrD* or *dnrF* resulted in activation of the entire examined CtrA regulon, regardless of oxygen tension. In *R. capsulatus* FnrL, in silico and ChIP-seq data also suggested regulation of the CtrA regulon, but it was only with loss of the redox regulator RegA where an actual transcriptional effect on the CtrA regulon was observed. For the first time, we show that there are complex interactions between redox regulators and the CtrA phosphorelays in these bacteria and we present several models for how these interactions might occur.

## 1. Introduction

Bacteria sense and process environmental signals in order to adapt to changes in their surroundings. These signals are relayed through regulatory networks that adjust the cells’ behavior, often through changes in gene expression. The alphaproteobacterium *Dinoroseobacter shibae* is a member of the marine roseobacter group and an aerobic anoxygenic photoheterotrophic bacterium, capable of both aerobic and anaerobic respiration [1]. It can be free-living or an algal symbiont [1] and is a metabolically versatile bacterium able to adapt to changes in its highly dynamic environment. For example, at the end of an algal bloom when the oxygen concentration drops, an alternative terminal electron acceptor such as nitrate can be used for respiration [1,2].

The response to the change from aerobic to anaerobic conditions is controlled by four Crp/Fnr transcriptional regulators in *D. shibae* [3]. Crp/Fnr regulators are widely distributed among bacteria and form a superfamily consisting of 14 phylogenetic subgroups [4]. The versatility of this family is reflected by both the wide range of signals that are sensed, such as temperature [5], oxygen [6], and nitric oxide (NO) [7], and the range of metabolic processes regulated upon activation, which include respiration-related processes and especially the transition between aerobic and anaerobic lifestyles [3,8].

Two well-studied members of this family are the Dnr and Fnr proteins. Dnr proteins bind a heme cofactor that allows for sensing of NO [4,9], while Fnr proteins react to low oxygen tension [4,6]. In *D. shibae*, FnrL and DnrD regulate DnrE and DnrF in a cascade-type network that controls the transition from aerobic to anaerobic growth, heme and carotenoid synthesis, multiple other metabolic processes, and flagellar synthesis [3]. The importance of these regulators in *D. shibae* is well illustrated by the observation that loss of FnrL affects the transcript levels of over 400 genes [3].

Another important regulatory system in *D. shibae* is the CtrA phosphorelay [10]. Like the Crp/Fnr regulators, this phosphorelay integrates an environmental signal, in this case, the autoinducer concentration as an indicator of cell density, and adjusts gene expression in response [11]. This phosphorelay is conserved within the majority of alphaproteobacterial lineages and consists of the histidine kinase CckA, the phosphotransferase ChpT and the transcriptional regulator CtrA [10]. In *D. shibae*, the CtrA phosphorelay is activated by the quorum sensing (QS) signal of the main acyl-homoserine lactone (AHL) synthase (LuxI_1_) with subsequent regulation of genes for flagellar motility, recombination and competence proteins, a tight adherence (tad) pilus involved in attachment to carbohydrates on the host cells [12], cell cycle control, gene transfer agent (GTA) production, bis-(3′-5′)-cyclic dimeric guanosine monophosphate (c-di-GMP) signaling, the NO-sensing heme-nitric oxide/oxygen binding domain (HNOX) protein, and the AHL synthases LuxI_2_ and LuxI_3_ [11,13,14]. Deletion of *cckA* has been found to abolish the mutualistic interaction between *D. shibae* and its algal host, demonstrating that the CtrA phosphorelay is essential for establishment of this symbiosis, at least partly due to the requirement for flagella [15]. The Crp/Fnr and CtrA phosphorelay networks are connected by their shared regulation of flagellar gene expression and due to their involvement in symbiosis with the host dinoflagellate.

There are three ways bacteria can be exposed to NO. Some bacteria generate NO during denitrification, and this is considered the activator for DnrD in *D. shibae* [3,16]. NO can be produced intracellularly through the oxidization of L-arginine to NO and L- citrulline [17] or via a nitric oxide synthase (NOS) [17,18]. NO released by some eukaryotic organisms can be a form of communication with their symbiotic bacteria and is then typically sensed by HNOX proteins [19]. The HNOX genes are often located adjacent to genes encoding c-di-GMP signaling proteins or histidine kinases. In the context of symbioses, only a few NO-detecting systems have been found that do not involve c-di-GMP signaling but instead directly integrate into QS systems [20,21,22]. In *D. shibae*, an HNOX protein detects NO and thereupon inhibits the c-di-GMP synthesizing enzyme Dgc1 [23].

The potential for overlap between Crp/Fnr-based regulation and the CtrA phosphorelay also exists in the purple non-sulfur alphaproteobacterium *Rhodobacter capsulatus*. Its CtrA phosphorelay was originally discovered due to its regulation of GTA production [24], but it also affects many other genes such as those associated with flagellar motility, gas vesicles, and c-di-GMP signaling [24,25]. Like *D. shibae*, *R. capsulatus* can switch between aerobic and anaerobic lifestyles, which involves Crp/Fnr regulation, the RegA/B two-component system, and CrtJ [26,27,28]. Loss of FnrL affects the transcript levels of 20% of *R. capsulatus* genes [29], including 42 that are directly regulated and encode c-di-GMP signaling, gas vesicle, and flagellar proteins, among others [29].

These initial surveys of the activities of redox regulators and the CtrA phosphorelays in *D. shibae* and *R. capsulatus* indicated a potential connection of the regulons. Therefore, we were interested in exploring in more detail the extent to which these regulatory systems interact. We re-analyzed ten available transcriptomic datasets for the two species. Deletion mutants, including those of redox regulators and the CtrA phosphorelay/QS networks, were analyzed to examine the regulon overlap of these systems and to evaluate their potential integration. We also included further analyses of available transcriptomic datasets of wild type strains undergoing physiological changes related to the environmental signals integrated by these regulatory systems.

## 2. Materials and Methods

### 2.1. Datasets Analyzed in this Study

Ten published and accessible microarray and RNA-seq transcriptomic datasets for chosen gene knockout strains and experiments monitoring responses to changes in environmental conditions were obtained from the NCBI GenBank database (Table 1).

### 2.2. Processing and Analysis of Datasets

This study includes four different types of transcriptomic data (Table 1) that could not be processed and analyzed as one dataset. We therefore used the changes in transcript levels (log_2_ fold change) compared to the controls used in the respective studies (e.g., wild type or time point before changes in the environmental conditions) for each dataset. RNA-seq data from *D. shibae* (reads per gene) and *R. capsulatus* (log_2_ fold change) were obtained from the respective publications (Table 1).

Agilent microarray datasets were processed using the LIMMA package in R [35]. Background correction was performed with the “normexp” method and an offset of 10. Two-color microarrays were normalized with the “loess” method before quantile normalization. Signals/intensities from spots were averaged.

Affymetrix microarray datasets were processed using the R packages LIMMA, makecdfenv, and affy [35,36,37]. The CDF environment for GSE18149 was generated using the corresponding CDF file downloaded from GEO (accession GPL9198). Data were normalized with the rma function. A linear fit model was generated for comparison.

In order to analyze the CckA and ChpT regulons, thresholds were set that allowed definition of regulated and non-regulated genes. These thresholds were applied to the log_2_ fold change in transcript level values in the *cckA* and *chpT* deletion mutants. A gene was not considered regulated when its log_2_ fold change was between 1 and −1 while a log_2_ fold change value above 1 or below −1 indicated an affected gene. The analyzed genes were grouped based on published information about their functional categories as described (Supplementary Table S1).

## 3. Results

### 3.1. Overlap of the Crp/Fnr and CtrA Regulons in *Dinorosebacter shibae*

**Figure 1:**
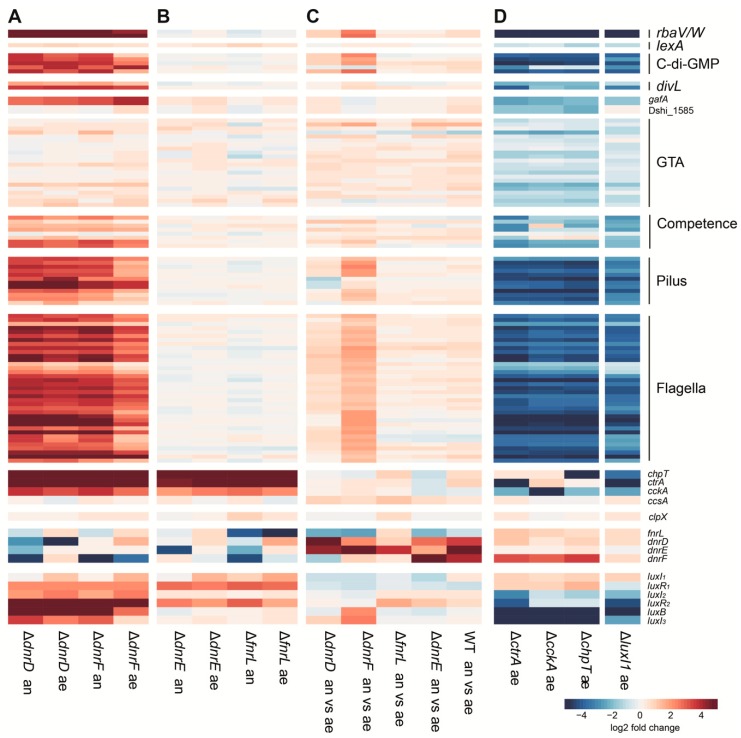
Transcriptomic data for genes in selected functional groups in different knockout strains. The four Crp/Fnr regulator knockouts were grown under aerobic (ae) or anaerobic (an) conditions. The log_2_ fold changes compared to the respective wild type (WT) (**A**,**B**) or against themselves grown at different conditions, are shown (**C**). The CtrA phosphorelay and quorum sensing system knockouts were grown aerobically to the stationary phase and compared to the WT (**D**). The functional group assignments on the right are based on published information as described in Supplementary Table S1. Note: the Δ*luxI_1_* strain retains a portion of the gene that can therefore result in mapped reads.

The possible interaction between the Crp/Fnr regulator and CtrA phosphorelay networks was first assessed using transcriptomic datasets for regulator deletion mutants. The changes of transcript levels of known Crp/Fnr- and CtrA-controlled traits revealed a strong overlap of both regulons, with the regulator-encoding genes themselves affected by losses of the other regulators (Figure 1). Under both aerobic and anaerobic conditions, the loss of *dnrD* or *dnrF* resulted in increased transcript levels of the CtrA phosphorelay, QS, flagellar motility, tad pilus, competence and recombination, gene transfer agent (GTA), *divL* and c-di-GMP signaling genes (Figure 1A). In all datasets, the GTA genes showed comparatively small changes in transcript levels (Figure 1), probably as a result of a small subpopulation actually expressing these genes [13]. Only the loss of *dnrF* led to a change in gene expression between aerobic and anaerobic conditions, since a greater increase in the transcript levels could be observed under anaerobic conditions for most of its regulon (Figure 1C). The loss of *fnrL* or *dnrE* resulted in increased transcript levels of *ctrA*, *cckA*, *chpT*, *luxI_1_*, *luxR_1_*, and *luxR_2_* but had little to no effect on the downstream CtrA regulon (Figure 1B).

Almost all examined genes showed an opposite pattern in the CtrA phosphorelay and *luxI_1_* mutants (Figure 1D) compared to *dnrD* and *dnrF* (Figure 1A). Most of the genes showed decreased transcript levels in strains lacking any of the CtrA phosphorelay genes, with the exceptions of the Crp/Fnr regulators where the largest increase was found for *dnrF* (Figure 1D). Loss of *luxI_1_* resulted in increased transcript levels for *fnrL*, *dnrD*, and *dnrF*, but no changes were observed for *dnrE* (Figure 1D).

### 3.2. The Role of ChpT in Signal Integration

In *D. shibae*, deletion of neither *ctrA* nor *cckA* had an influence on expression of *chpT*, whereas the loss of either *ctrA* or *chpT* resulted in decreased expression of *cckA* (Figure 1D) [11]. However, all three CtrA phosphorelay component genes showed reduced transcript levels in the absence of the AHL synthase *luxI_1_* (Figure 1D) [13], whereas loss of the Crp/Fnr regulators resulted in increased transcript levels of these genes (Figure 1A,B). Therefore, in contrast to *ctrA* and *ccka*, *chpT* is not regulated by the CtrA phosphorelay itself, but by other factors that can thereby control the phosphorylation state of CtrA. These findings also suggest that *chpT* transcription is regulated oppositely by QS and the Crp/Fnr regulators.

This is supported by binding site predictions for FnrL [3] that suggest it binds at the promoter of *chpT* and *clpX*, which encodes a protease known to cleave CtrA [3,38,39]. Deletion of *fnrL* strongly increased the expression of *chpT* but only resulted in minimal changes for *clpX* (Figure 1B). Binding site prediction for the Dnr regulators did not find any binding sites near *clpX* or the CtrA phosphorelay genes [3].

**Figure 2:**
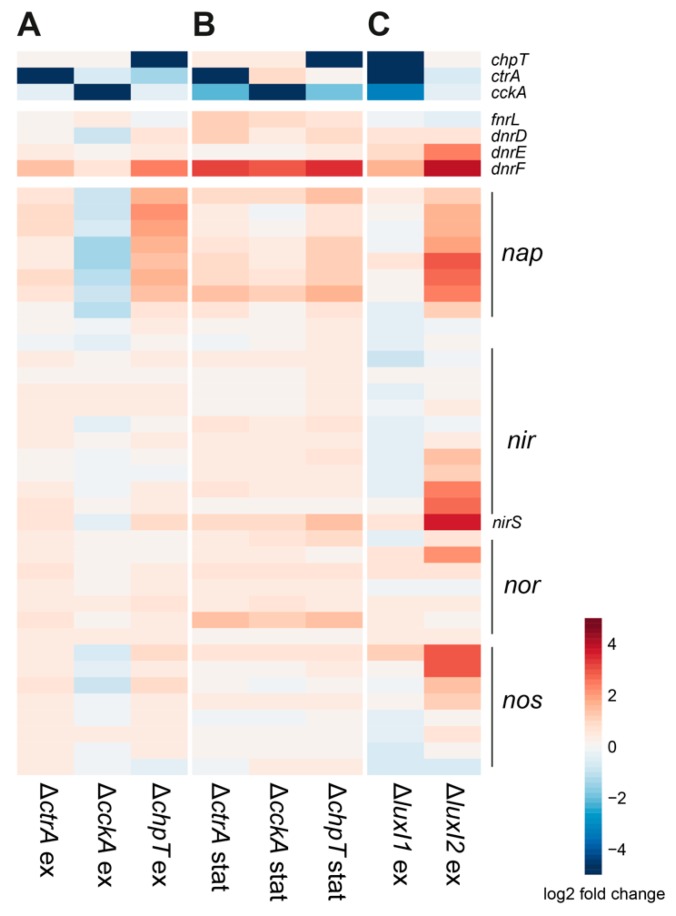
Comparison of CtrA phosphorelay, Crp/Fnr regulator, and denitrification gene expression control by CtrA phosphorelay and LuxI_1/2_ synthases during exponential and stationary growth phases. Samples for the *ctrA*, *cckA*, and *chpT* knockout mutants were analyzed at mid-exponential (OD 0.4) (**A**) and stationary (six hours after onset of stationary phase) (**B**) phases of growth. The Δ*luxI_1_* data were obtained during stationary phase, six hours after the onset of stationary phase, and the Δ*luxI_2_* data were obtained during the mid-exponential growth phase (**C**).

It was previously found that more genes were affected by the loss of *chpT* than *cckA* [11], suggesting ChpT regulates some genes independent of CckA and that a different kinase might regulate its activity and thereby affect downstream gene expression. Among the genes affected by the loss of *chpT* but not *cckA*, *dnrF* was the most upregulated gene during exponential growth while *lexA* and *recA* were among those most downregulated genes in both exponential and stationary phases (Figure 2). Although there was a small increase in transcript levels of *dnrF* in the *cckA* deletion strain during exponential growth, it did not pass the threshold we defined (see Materials and Methods). These findings suggest a link between *dnrF* and *chpT*.

Additional discrepancies between CckA and ChpT are apparent from their opposing effects on the *nap* gene cluster during exponential growth (Figure 2A), although this is not maintained in stationary phase (Figure 2B). In exponential phase, loss of *cckA* led to decreased transcript levels of the *nap* gene cluster, while the loss of *ctrA* and *chpT* led to increased levels (Figure 2A). This cluster is the only denitrification cluster activated by FnrL but repressed by the three Dnr regulators [3]. Interestingly, transcript levels of all four denitrification gene clusters were increased in the AHL synthase knockout Δ*luxI_2_* but were unaffected in Δ*luxI_1_* (Figure 2C).

### 3.3. Time-Resolved Evaluation of Environmental Changes and the Regulation of c-di-GMP Signaling Genes

**Figure 3:**
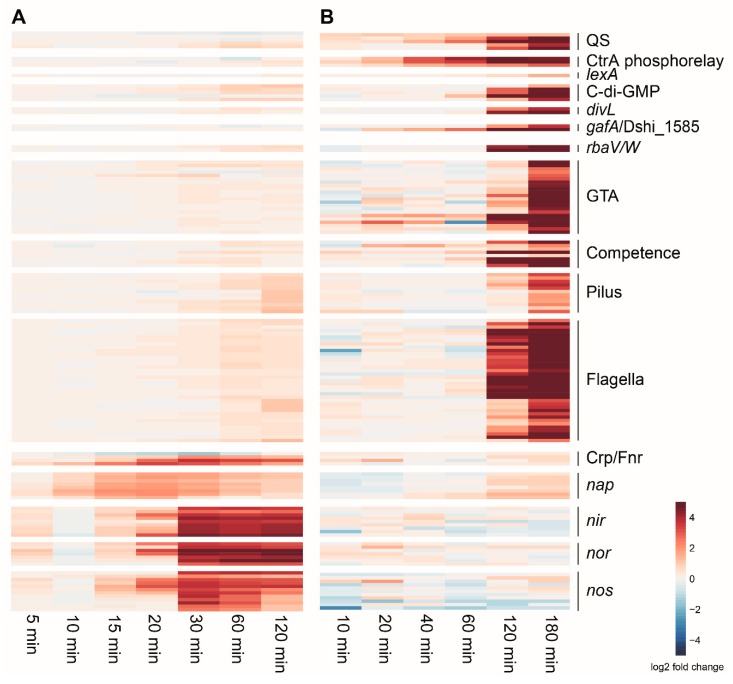
Time-resolved transcriptomic analysis for genes in selected groups in response to environmental changes. (**A**) Gene expression changes after the shift to anaerobic growth compared to aerobic conditions. (**B**) Gene expression after external addition of autoinducer 3-oxo C14 HSL to the QS synthase null mutant (Δ*luxI_1_*).

**Figure 4:**
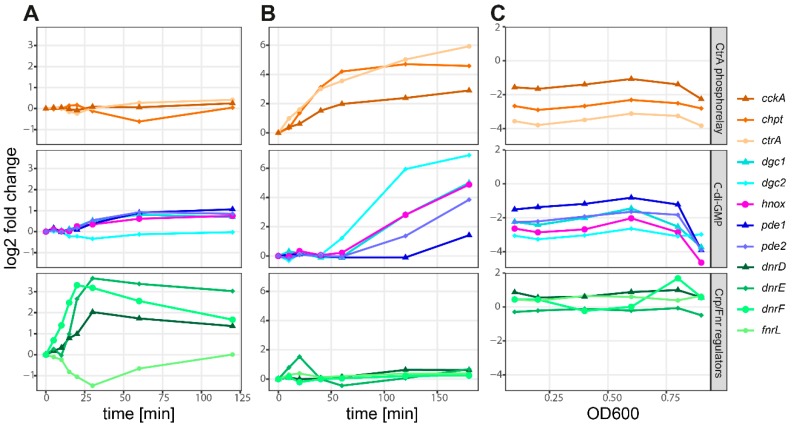
Time- and density-resolved transcript levels in three different conditions for three groups of regulators. The expression profiles of the CtrA phosphorelay genes (top), c-di-GMP signaling genes (middle), and four Crp/Fnr regulator-encoding genes (bottom) are plotted. The changes in transcript levels were monitored after the switch from aerobic to anaerobic growth over a time period of 120 min (**A**), after the external addition of autoinducer (3-oxo C14 HSL) to the synthase null mutant (Δ*luxI_1_*) over a period of 180 min (**B**), and during logarithmic (samples 1–5) and stationary (sample 6) phases of growth as determined by optical density (**C**).

Interactions between the networks in *D. shibae* were further analyzed using time-resolved transcriptomic datasets. These were collected following the switch from aerobic to anaerobic conditions in wild type cells (Figure 3A and Figure 4A) [31], following the external addition of AHL autoinducer to the AHL synthase mutant Δ*luxI_1_* (Figure 3B and Figure 4B) [13], and through the culture growth phases for Δ*luxI_1_* in the absence of AHL (Figure 4C) [14].

Upon the shift to anaerobic conditions, all three *dnr* genes showed an immediate increase in transcript levels for 30 min and then stayed constant, whereas those of *fnrL* decreased (Figure 4A). These changes corresponded with increased transcript levels of the denitrification gene clusters, with the *nap* cluster showing a slightly different pattern than the *nir* and *nos* clusters (Figure 3A). Slight increases were observed for the c-di-GMP signaling, flagellar, tad pilus, and QS genes (Figure 3A). Four of the five c-di-GMP signaling genes showed increased transcript levels following the transfer to an anaerobic environment, whereas *dgc2* showed a slight decrease (Figure 4A).

The addition of AHL to the Δ*luxI_1_* strain led to increased transcript levels for all CtrA- and QS-controlled genes (Figure 3B). This included the CtrA phosphorelay and c-di-GMP signaling genes, with *dgc2* showing the largest increase (Figure 4B). No effect was visible for the Crp/Fnr regulator-encoding genes (Figure 4B) and only a minor increase of the *nap* gene cluster was observed among the denitrification genes (Figure 3B).

Due to the increased transcript levels observed for CtrA regulon genes in the *dnrD* and *dnrF* deletion strains, it was expected that the same genes would also be decreased under anaerobic conditions. Instead, it turned out that the change from aerobic to anaerobic conditions (Figure 3) resulted in increased transcript levels for these genes. However, this increase was small, and effects were not observed for some genes that appeared to be controlled by the individual regulators based on the knockout transcriptomic data (Figure 1). This included the regulation of the CtrA phosphorelay genes by the Crp/Fnr regulators. Vice versa, loss of the CtrA phosphorelay genes indicated their repression of Crp/Fnr regulator gene expression (Figure 1D), but the contrary was observed in the respective physiological datasets where the Crp/Fnr regulators seem to be upregulated (Figure 3B). Notably however, in both physiological datasets, *dgc2* stands out as distinctly affected compared to other c-di-GMP signaling genes (Figure 4A,B). Also, in the non-induced Δ*luxI_1_* culture, no influence of the QS null mutant on the Crp/Fnr regulators was observed, but the CtrA phosphorelay and c-di-GMP signaling genes were down-regulated (Figure 4C).

Interestingly, in contrast to *fnrL*, *dgc2*, and *chpT*, the other Crp/Fnr regulators, c-di-GMP signaling, and CtrA phosphorelay genes all decreased at the onset of the stationary phase (Figure 4C). Moreover, analysis of the Crp/Fnr knockout data showed that the loss of *dnrF* or *dnrD* resulted in increased transcript levels of four of the c-di-GMP signaling genes under anaerobic growth conditions, with only *dgc2* being unaffected (Figure S1A). Loss of *luxI_1_* and the CtrA phosphorelay genes resulted in decreased transcripts for all five genes (Figure S1B,C), although the effects on *dgc2* were smaller than for the other genes in the stationary phase (Figure S1C).

### 3.4. Effects on the CtrA Regulon during Coculture of Dinoroseobacter shibae and Its Algal Host

In the two-phase interaction of *D. shibae* with its dinoflagellate host *Prorocentrum minimum* [14,40], a mutualistic growth phase (0 to 21 days of cocultivation) is followed by a pathogenic growth phase (21 to 30 days of cocultivation) that leads to death of the algae [15]. Analysis of the transcriptomic data of *D. shibae* during cocultivation showed an overall increase in the transcription for the CtrA regulon genes during the transition between the two phases (day 24 compared to day 18), followed by a decrease during the late-pathogenic phase, after 30 days (Supplementary Figure S2).

Of the CtrA phosphorelay genes, only *chpT* remained upregulated during the pathogenic interaction. Evaluation of the denitrification gene clusters showed strong variation among these genes (Supplementary Figure S2), likely arising from overall low expression levels, and this made it difficult to draw any conclusions.

### 3.5. RegA Activates the CtrA Regulon in Rhodobacter capsulatus

Next, we asked if the observed overlap between redox regulators and the CtrA phosphorelay system is conserved in another member of the family *Rhodobacteraceae*. For *R. capsulatus*, transcriptomic data were available for knockout mutants of *ctrA*, *cckA*, and the known redox regulator-encoding genes *fnrL*, *regA*, and *crtJ*. We identified three additional Crp/Fnr regulator-encoding genes in this bacterium based on blast searches (RCAP_rcp00107, RCAP_rcc01561, RCAP_rcc03255), but these genes showed no evidence of differential regulation in any of the analyzed datasets and we did not consider them further. A blast search also identified a homologue (RCAP_rcc02630) of the HNOX-encoding gene of *D. shibae* (Dshi_2815). This gene encodes a protein with a predicted heme nitric oxide/oxygen binding (HNOB) domain and is located adjacent to a c-di-GMP signaling gene (RCAP_rcc02629) that was recently demonstrated to affect GTA production and motility in *R. capsulatus* [41]. When bound to NO, the HNOX homologue in *D. shibae* inhibits the activity of the diguanylate cyclase Dgc1, which is encoded by the neighboring gene [23].

**Figure 5:**
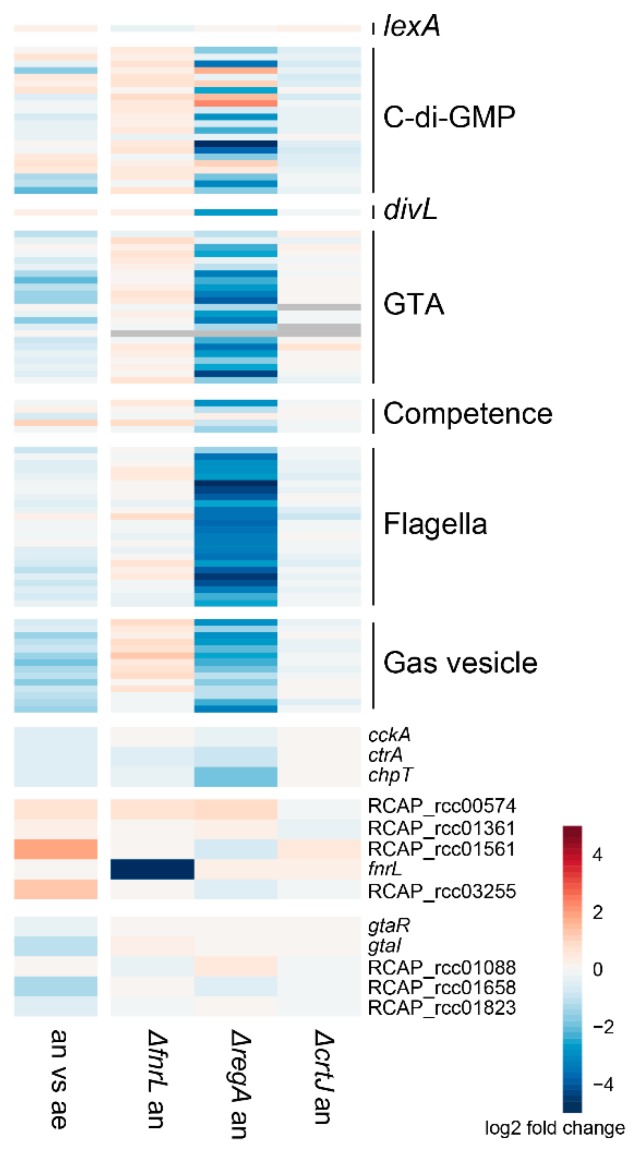
Effects of growth conditions and three regulator knockouts on the transcript levels of eight categorized groups of genes in *Rhodobacter capsulatus*. The microarray-based transcriptomic data for aerobic versus anaerobic growth in the wild type and for three mutants, *fnrL*, *regA*, and *crtJ*, compared to the wild type are shown.

FnrL is the only Crp/Fnr regulator that has been studied in *R. capsulatus* [29]. Its loss did not result in any large changes in transcript levels for the examined traits under anaerobic phototrophic conditions (Figure 5), and the same was observed for the loss of *crtJ*, which encodes a transcription factor that controls numerous photosynthesis and cytochrome genes [32] (Figure 5). RegA is the response regulator of the RegB/A two-component system that controls photosynthesis, nitrogen and carbon fixation, denitrification, and respiration genes in response to oxygen availability [26]. In contrast to *fnrL* and *crtJ*, we found that the loss of *regA* resulted in a strong decrease in transcript levels of the CtrA regulon genes (Figure 5), indicating that RegA acts as a direct or indirect activator of these genes. Like the genes involved in regulation of photosynthesis and the change between aerobic/anaerobic lifestyle in *D. shibae*, loss of *regA* affected *chpT* the most among the CtrA phosphorelay genes in *R. capsulatus*. Loss of the CtrA phosphorelay genes had no influence on transcription of *fnrL*, *regA*, *regB*, or the other putative Crp/Fnr regulator-encoding genes (Supplementary Figure S3). A comparison of photosynthetic anaerobic growth and aerobic cultivation in *R. capsulatus* showed the CtrA-regulated traits have reduced transcript levels under anaerobic conditions (Figure 5).

## 4. Discussion

### 4.1. The Crp/Fnr and CtrA/QS Regulons Overlap in Dinoroseobacter shibae

Our analysis revealed an inverse regulatory crosstalk between the Crp/Fnr and CtrA systems in *D. shibae*. We found the denitrification gene clusters and Crp/Fnr regulator genes, especially *dnrF*, to be part of the CtrA phosphorelay and LuxI_2_ regulons. *DnrE* was affected exclusively by loss of LuxI_2_, whereas loss of LuxI_1_ only had minor effects on *fnrL*, *dnrD*, and *dnrF* and no effect on *dnrE*. In addition to their regulation by LuxI_1_, which signals cell density, the Crp/Fnr regulators integrate oxygen and NO levels and affect all three CtrA phosphorelay genes.

Until now, overlapping regulation by the Crp/Fnr and CtrA systems has only been noted in *D. shibae* for flagellar genes and *recA* [3,12,13], and to our knowledge this level of regulatory interaction has not been reported for alphaproteobacteria. However, a comparable connection between QS and Crp/Fnr regulators has been documented for the gammaproteobacterium *Pseudomonas aeruginosa* where the regulons of the FnrL homolog Anr and QS synthase LasR overlap. Here, denitrification genes are induced by Anr and inhibited by LasR. Additionally, in the absence of *lasR*, Anr regulates production of the QS molecule 4-hydroxy-2-alkylquinoline [42]. At the protein level, nitrite reductase (NirS), a flagellar protein (FliC), and the chaperone DnaK form a complex that influences flagellar formation and motility and thus creates a link between denitrification and motility [43]. In cystic fibrosis infections, *P. aeruginosa* is exposed to ambient conditions with low oxygen tension. The intracellular levels of c-di-GMP increase, which leads to biofilm formation. These conditions also lead to an increase in mutations in the QS transcriptional regulator-encoding gene *lasR*. As *lasR* deletion strains grow to higher cell densities and have higher denitrification rates, it has been suspected that these mutations increase the fitness of the population during infection [44,45,46].

Combined, these observations indicate that there may be a more widely conserved interaction of Crp/Fnr regulators and QS in proteobacteria. The CtrA phosphorelay is unique to alphaproteobacteria, indicating a potential independent evolution of this regulatory crosstalk in this lineage.

### 4.2. Inverse Control of the CtrA Regulon by RegA and Anaerobic Photosynthetic Growth Conditions in Rhodobacter capsulatus

In *R. capsulatus*, the regulons of the redox-responsive two-component system RegA/B [47] and the CtrA phosphorelay overlap. Interestingly, *chpT* stands out because it is the only CtrA phosphorelay gene that is regulated by RegA. Similar to Dnr and Fnr in *D. shibae*, RegA controls the expression of photosynthesis and respiration genes [26]. ChIP-seq with RegA identified binding sites adjacent to several genes also targeted by CtrA: RCAP_rcc02857 (a c-di-GMP signaling gene involved in GTA production) and its divergently transcribed neighbor (RCAP_rcc02856), RCAP_rcc02683 (a chemotaxis gene), and *dksA* (a *dnaK* suppressor gene) [34].

As in *D. shibae*, transcriptomic data from a *fnrL* deletion strain showed no effects on the CtrA-controlled traits outside of the CtrA phosphorelay genes themselves. However, ChIP-seq and in silico binding site predictions [29] suggest FnrL binding adjacent to *divL*, *dnaK*, *recA*, flagellar gene clusters, the RcGTA capsid protein-encoding gene, and c-di-GMP signaling genes (including those affecting RcGTA production [41]). Similarly, ChIP-seq with CrtJ [48], a regulator controlling expression of multiple genes involved in photosynthesis, also revealed a connection to the CtrA phosphorelay. Even though the observed transcript level changes in the *crtJ* mutant were small, binding was found adjacent to *ctrA*, *clpX*, a *luxR* family gene*, dnaA*, *spoT*, *ftsZ*, and the first gene in the GTA structural gene cluster (RCAP_rcc01682) under aerobic and anaerobic cultivation. Binding sites adjacent to *dnaK* and two flagellar genes (*flgB* and *flaA*) were identified under aerobic and anaerobic conditions, respectively.

In *D. shibae*, deletions of the Crp/Fnr regulator-encoding genes indicated an inhibition of the CtrA regulon, but the physiological changes detected by these regulators (switch from aerobic to anaerobic conditions) showed a tendency towards activation of the CtrA regulon. The same was observed for the deletion mutants of the CtrA phosphorelay components and their regulation of the Crp/Fnr regulator genes. In *R. capsulatus*, we could observe a similar pattern but in reverse for regulation of the CtrA regulon by RegA. While the *regA* knockout indicated activation of the CtrA regulon, the switch to anaerobic photosynthetic growth conditions showed an inhibition. This is probably indicative of a more complex interaction among these regulatory systems. However, the *regA* deletion transcriptomic data are supported by in vivo motility tests that showed reduced swimming ability of the Δ*regA* strain [26].

### 4.3. Integration of Crp/Fnr Regulation into the CtrA Phosphorelay and Regulon

**Figure 6:**
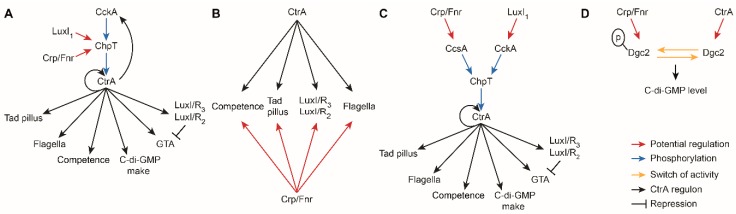
Possible mechanisms of integration of the Crp/Fnr and CtrA systems. (**A**) The LuxI_1_ and Crp/Fnr signals could be integrated into the CtrA phosphorelay via *chpT* regulation, which does not happen via CckA or CtrA. (**B**) Shared binding site motifs for Crp/Fnr regulators and CtrA might allow direct integration of the NO/oxygen signal into the CtrA regulon. (**C**) An additional histidine kinase (CcsA) has been reported to phosphorylate ChpT in another bacterium, and this could integrate the Crp/Fnr signals and disconnect CckA from the integration. (**D**) Phosphorylation of the Dgc2 receiver domain likely regulates the enzyme’s diguanylate cyclase activity and thereby alters the intracellular levels of c-di-GMP, which are known to affect the CtrA regulon.

In *D. shibae*, CtrA binding site predictions and expression data for *ctrA* and *cckA* suggest that CtrA directly regulates its own expression and that of *cckA*, but not *chpT* [13]. Therefore, *chpT* transcription must be regulated from outside of the CtrA phosphorelay and upstream of CtrA. Both, regulatory control of *chpT* and signal integration upstream of CtrA is known for LuxI_1_ [11]. A similar situation might be possible for Crp/Fnr signal integration due to their regulation of *chpT* (Figure 6A). Since *chpT* is the only RegA-regulated CtrA phosphorelay gene in *R. capsulatus* (and it has a RegA binding site), it seems to play a central role here, too. However, there are also RegA binding sites associated with *clpX* and other genes of the CtrA regulon [26]. Interestingly, the Dnr/Fnr binding site in the *nosR2* promoter in *D. shibae* has the sequence 5′-TTAAC-N4-GTCAA-3′ [3], which shares a half-site binding motif with CtrA 5′-TTAAC-N5-GTTAAC-3′ [11]. Previously, comparison between transcriptional regulation and the presence of full and half-site motifs revealed the potential importance of half-site motifs for transcriptional control by CtrA in *R. capsulatus* [34]. Thus, CtrA and Fnr regulators might interact with some of the same/overlapping sequences (Figure 6B).

A distinct role for ChpT is supported by the observation that loss of *chpT* or *ctrA* but not *cckA* results in decreased transcript levels of *dnrF*. It is possible that ChpT integrates signals from more than one kinase into its regulation of CtrA. To our knowledge, the only other instance of a histidine kinase affecting phosphorylation of CtrA via ChpT is CcsA from *Sphingomonas melonis* [49]. Potential homologues of CcsA are encoded in *D. shibae* (Dshi_1893) and *R. capsulatus* (RCAP_rcc02545), but effects on transcript levels of these genes were not observed in any of the analyzed datasets. This does not exclude their involvement but also does not allow us to draw further conclusions (Figure 6C).

### 4.4. Crp/Fnr Regulation of the CtrA Regulon is Largely Independent of Oxygen Tension

Among the Crp/Fnr regulators, only loss of the NO-sensing DnrF resulted in higher inhibition activity of the CtrA system under anaerobic conditions. In *P. aeruginosa*, swimming motility is controlled anaerobically and aerobically and it was suggested that NirS promotes motility in multiple ways, at the protein level or via signaling pathways, depending on oxygen availability [50]. Regulation of QS traits by both NO and oxygen was also found in the interaction of *Vibrio fischeri* with the light organ of its squid host. Here, NO released by the host’s immune system regulates the symbionts’ settlement via biofilm production while the host’s control of oxygen availability regulates bacterial bioluminescence in a circadian manner [51,52,53]. However, since the Crp/Fnr knockout and physiological change transcriptomic data have opposite effects on the CtrA phosphorelay (inhibition indicated by the knockouts and activation by the shift to anaerobic growth), it is difficult to determine the role of oxygen on the CtrA phosphorelay. In *R. capsulatus*, the knockout transcriptomic data were supported by in vivo experiments, so if the knockout transcriptomic data also reflect the actual CtrA regulon in *D. shibae*, the Crp/Fnr regulators have an inhibitory effect on the CtrA phosphorelay and its regulon. It is known that *Dinoroseobacter* establishes a mutualistic symbiosis with its dinoflagellate host via the CtrA phosphorelay and by means of flagella. It is possible this interaction is repressed towards the end of an algal bloom when oxygen concentrations change, resulting in downregulation of flagella (and other CtrA-regulated traits) via Crp/Fnr regulation.

### 4.5. The Role of c-di-GMP

Multiple eukaryotic hosts are known to use NO for communication with microbial symbionts. In some of the characterized systems, NO is sensed by HNOX proteins, which then control c-di-GMP signaling proteins or histidine kinases encoded by genes adjacent to the HNOX-encoding gene. For example, in *Vibrio harveyi*, the HNOX-neighboring histidine kinase phosphorylates the QS transcription regulator LuxU [20], and in *D. shibae*, HNOX inhibits the c-di-GMP signaling enzyme Dgc1 [23]. However, *D. shibae* also has a second c-di-GMP synthesizing enzyme, Dgc2. During adaptation to anaerobic cultivation and at the onset of stationary phase, *dgc2* transcriptional patterns were similar to *chpT* and *fnrL*. The transcript levels of these three genes plateaued, whereas those of the other c-di-GMP signaling, CtrA phosphorelay and Crp/FnrL genes decreased. A unique regulation of *dgc2* was also observed in the *dnrF*, *dnrD*, *cckA*, and *chpT* knockout strains. Thus, both networks (Crp/Fnr and CtrA phosphorelay) regulate *dgc2* and affect its expression in a similar manner as a response to the onset of stationary phase.

The role of *dgc2* in the CtrA phosphorelay and FnrL networks and how it might connect both remain to be clarified. For example, it is possible that phosphorylation of the receiver domain of Dgc2 regulates its c-di-GMP synthase activity. As a result, regulation by the Crp/Fnr or CtrA phosphorelay systems could have different effects on the shared traits (Figure 6D).

## 5. Conclusions

In this study we show that regulation of the CtrA regulon, including traits related to phenotypic heterogeneity, is additionally controlled by the aerobic–anerobic regulators Crp/Fnr in *D. shibae* and by FnrL/RegA in *R. capsulatus*. This finding is especially important for the understanding of the metabolically flexible lifestyles of these bacteria. The analysis of the available transcriptomic datasets revealed multiple possible integration sites of the Crp/Fnr signal into the CtrA phosphorelay, but a final explanation is still elusive based on these data. Nevertheless, this investigation provides the first insights into the integration of a second environmental signal into the CtrA phosphorelay and demonstrates a strong transcriptional connection between QS, CtrA-regulated traits, and Crp/Fnr regulators in alphaproteobacteria, which has an interesting parallel with QS and Crp/Fnr regulators in a second class of proteobacteria. To our knowledge, *D. shibae* and *R. capsulatus* are the first two organisms where both Dnr and HNOX NO-sensor proteins have been studied. Further investigation is necessary to clarify the interaction between the CtrA phosphorelay and the Crp/Fnr regulators. For example, it would be helpful to confirm if an additional kinase is indeed regulating ChpT in these bacteria.

## Figures and Tables

**Table 1:** Description of the transcriptomic datasets analyzed in this study.

Species	Strains and Culture Conditions	Type of Data	Accession Number	Reference
*D. shibae*	Time-resolved response to addition of AHL to Δ*luxI_1_*	RNA-seq	GSE122111	[13]
	Time resolved co-cultivation with *Prorocentrum minimum*	RNA-seq	GSE55371	[15]
	Knockouts of *ctrA*, *chpT*, and *cckA* in exponential and stationary phases of growth	Agilent dual-color microarray	GSE47451	[11]
	Knockouts of *fnrL*, *dnrD*, *dnrE*, and *dnrF* under aerobic conditions and 60 min after shift to anaerobic, denitrifying conditions	Agilent dual-color microarray	GSE93652	[3]
	Time-resolved growth of wild type and Δ*luxI_1_* strains from OD_600_ 0.1 to stationary phase	Agilent dual-color microarray	GSE42013	[14]
	Δ*luxI_2_* growth to OD_600_ of 0.4	RNA-seq	PRJEB20656	[30]
	Time-resolved shift of the wild type from aerobic to anaerobic growth conditions	Agilent single-color microarray	GSE47445	[31]
*R. capsulatus*	Knockouts of *regA*, *crtJ*, and *fnrL* in mid-exponential growth phase	RNA-seq	PRJNA357604	[32]
	Knockouts of *ctrA* and *cckA* in mid-exponential growth phase	Affymetrix microarray	GSE53636	[33]
	Knockout of *ctrA* during exponential and stationary growth phases	Affymetrix microarray	GSE18149	[34]

## Supplementary Materials

The following are available online at https://www.mdpi.com/2076-2607/8/4/562/s1: Figure S1. Transcript level changes of *D. shibae* c-di-GMP signaling genes; Figure S2. Comparison of changes in transcript levels during different stages of the “Jekyll and Hyde” interaction between *Dinoroseobacter shibae* and the dinoflagellate *Prorocentrum minimum*; Figure S3. Transcript level changes of various FnrL- and RegA-related genes in CtrA phosphorelay mutants during exponential and stationary phases of growth in *Rhodobacter capsulatus*; Table S1. Assignment of genes into functional categories.
