# Supplementary material for: Interactions among Redox Regulators and the CtrA Phosphorelay in Dinoroseobacter shibae and Rhodobacter capsulatus
Source: Microorganisms. 2020 Apr 14;8(4):562. doi: 10.3390/microorganisms8040562 (PMC7232146; doi:10.3390/microorganisms8040562)
Supplement: Supplementary file 1 [file microorganisms-08-00562-s001.zip › microorganisms-756869-supplementary - Copy/Supplementary Figures.docx]

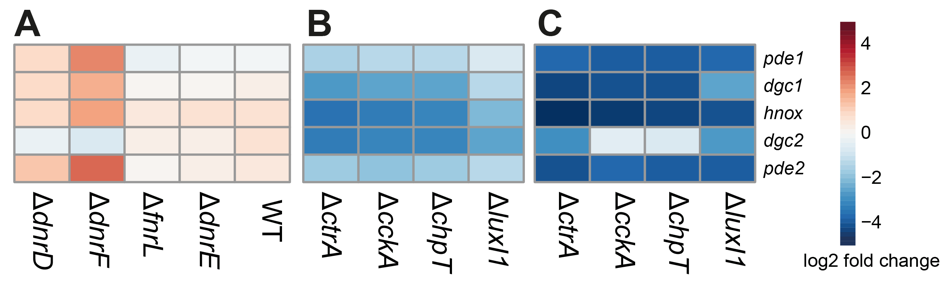


Figure S1. Transcript level changes of *D. shibae* c-di-GMP signaling genes. The transcript levels of the c-di-GMP-related genes was observed in four Crp/Fnr mutants and the wild type under anaerobic growth conditions as compared to aerobic growth (**A**), and in knockout strains of *luxI_1_* and the CtrA phosphorelay components during logarithmic (**B**) and stationary (**C**) phases of growth compared to the wild type.


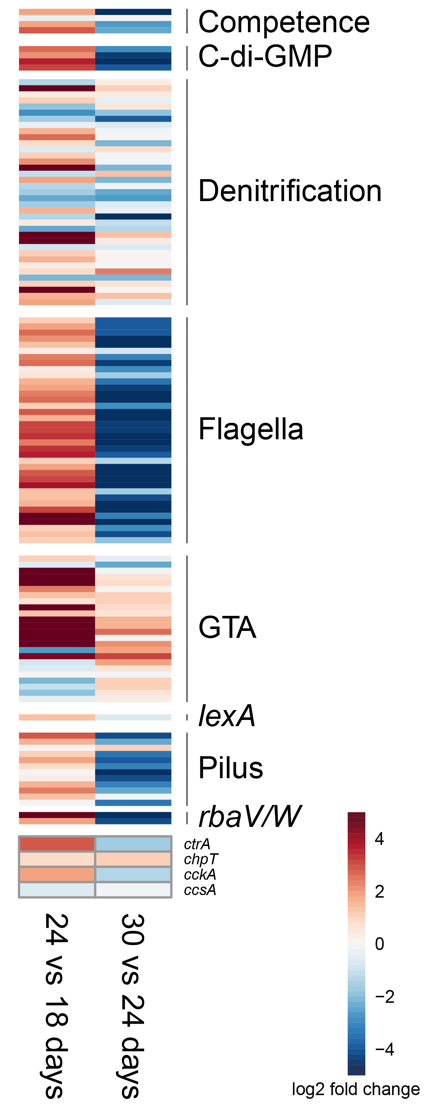


Figure S2. Comparison of changes in transcript levels during different stages of the “Jekyll and Hyde” interaction between *Dinoroseobacter shibae* and the dinoflagellate *Prorocentrum minimum*. Changes in transcript levels for selected gene groups in *D. shibae* during the mutualistic (18 days) relative to transition (24 days) phases and transition relative to pathogenic (30 days) phases of cocultivation with *P. minimum* are shown.


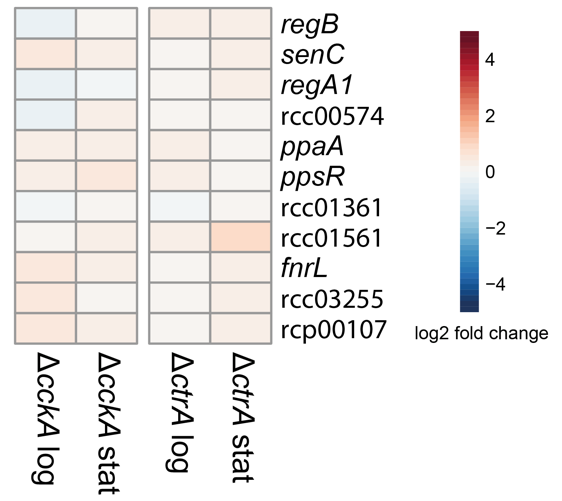


Figure S3. Transcript level changes of various FnrL- and RegA-related genes in CtrA phosphorelay mutants during exponential and stationary phases of growth in *Rhodobacter capsulatus.*
